# A Novel Transfer Learning‐Based Hybrid EEG‐fNIRS Brain‐Computer Interface for Intracerebral Hemorrhage Rehabilitation

**DOI:** 10.1002/advs.202505426

**Published:** 2025-08-19

**Authors:** Danyang Chen, Jian Shi, Bo Tao, Xingwei Zhao, Zhixian Zhao, Shengjie Li, Yeguang Xu, Tao Ding, Peng Zhang, Qing Ye, Kuiyou Chen, Zhuojin Wu, Yingxin Tang, Wei Jiang, Kai Shu, Li Huang, Zheng You, Ping Zhang, Zhouping Tang

**Affiliations:** ^1^ Department of Neurology Tongji Hospital, Tongji Medical College Huazhong University of Science and Technology Wuhan Hubei 430030 China; ^2^ State Key Laboratory of Intelligent Manufacturing Equipment and Technology Huazhong University of Science and Technology Wuhan Hubei 430074 China; ^3^ Hubei Bioinformatics and Molecular Imaging Key Laboratory Department of Biomedical Engineering, College of Life Science and Technology Huazhong University of Science and Technology Wuhan Hubei 430074 China; ^4^ Big Data and Artificial Intelligence Office, Tongji Hospital, Tongji Medical College Huazhong University of Science and Technology Wuhan Hubei 430030 China; ^5^ College of Computer Science and Technology Donghua University Shanghai 201620 China; ^6^ Department of Neurosurgery Tongji Hospital Tongji Medical College Huazhong University of Science and Technology Wuhan Hubei 430030 China; ^7^ Wuhan Neuracom Technology Development Co., Ltd Wuhan Hubei 430206 China; ^8^ Microsystems Technology Center School of Mechanical Science and Engineering Huazhong University of Science and Technology Wuhan Hubei 430074 China

**Keywords:** brain‐computer interfaces, electroencephalograph, functional near infrared imaging, intracerebral hemorrhage, motor imagery, transfer learning

## Abstract

Motor imagery (MI)‐based neurorehabilitation shows promise for intracerebral hemorrhage (ICH) recovery, yet conventional unimodal brain‐computer interfaces (BCIs) face critical limitations in cross‐subject generalization. This study presents a multimodal electroencephalography (EEG)‐functional near‐infrared spectroscopy (fNIRS) fusion framework incorporating a Wasserstein metric‐driven source domain selection method that quantifies inter‐subject neural distribution divergence. Through comparative neuroactivation analysis of 17 normal controls and 13 ICH patients during MI tasks, the transfer learning model achieved 74.87% mean classification accuracy on patient data when trained with optimally selected normal templates. Cross‐validation on two public hybrid EEG‐fNIRS datasets demonstrated generalizability, increasing baseline accuracy to 82.30% and 87.24%, respectively. The proposed system synergistically combines the millisecond temporal resolution of EEG with the hemodynamic spatial specificity of fNIRS, establishing the first clinically viable multimodal analytical protocol for ICH rehabilitation. This paradigm advances neurotechnology translation by paving the way for personalized rehabilitation regimens through robust cross‐subject neural pattern transfer while addressing the critical barrier of neurophysiological heterogeneity in post‐ICH populations.

## Graphical abstract

The study has developed the first clinically validated hybrid EEG‐fNIRS brain‐computer interface fusion framework that systematically overcomes cross‐subject generalization barriers through a novel Wasserstein metric‐driven neural template selection mechanism. By quantitatively mapping neural distribution divergences between 17 normal controls and 13 intracerebral hemorrhage patients during motor imagery tasks, this multimodal system achieves 74.87% patient‐specific classification accuracy via optimal normal template transfer.

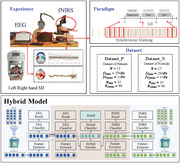

## 1 Introduction

Stroke remains a leading global cause of long‐term disability,^[^ ^1^ ^]^ with 55–75% of survivors experiencing upper‐limb motor impairments that reduce functional independence and socioeconomic well‐being.^[^ ^2^, ^3^, ^4^ ^]^ Intracerebral hemorrhage (ICH), comprising 6.5–19.6% of all stroke cases worldwide yet accounting for over 40% of stroke‐related deaths, poses unique neurorehabilitation challenges due to severe corticospinal tract damage and limited recovery.^[^ ^5^, ^6^ ^]^ Traditional rehabilitation paradigms demonstrate limited efficacy in addressing post‐ICH motor deficits, particularly in upper‐limb` functional restoration‐a critical determinant of quality‐of‐life recovery. Despite these challenges, brain‐computer interface (BCI) technology emerges as a promising neuromodulatory approach, capable of bypassing damaged neural pathways by directly decoding motor intention signals from preserved cortical activity.

BCIs establish direct neurophysiological communication channels by decoding cortical activity independently of peripheral neuromuscular pathways.^[^ ^7^ ^]^ This approach has emerged as a promising neuromodulatory intervention for post‐ICH rehabilitation. Neuroimaging evidence confirms that motor imagery (MI) activates sensorimotor networks akin to those engaged during real movement,^[^ ^8^ ^]^ enabling two major therapeutic pathways in ICH recovery: 1) functional compensation via neuroprosthetic control of assistive devices (e.g., robotic exoskeletons or virtual interfaces) based on decoded motor intentions,^[^ ^9^, ^10^, ^11^ ^]^ and 2) cortical reorganization guided by Hebbian plasticity‐driven sensorimotor circuit remodeling with repeated MI training.^[^ ^12^ ^]^ While MI‐BCI exhibits transformative potential, its clinical impact hinges on three interdependent factors: robust signal acquisition, precise feature extraction of pathological neural signatures, and high‐accuracy classification‐ all of which pose significant technical challenges in present‐day applications.

MI‐BCIs are categorized as invasive or non‐invasive based on their neural signal acquisition methodologies, with non‐invasive systems dominating clinical usage due to their advantages in safety and tolerability.^[^ ^13^, ^14^ ^]^ Electroencephalography (EEG), the most widely employed noninvasive modality, records cortical electrophysiological activity via scalp electrodes with millisecond temporal resolution. Despite its portability and suitability for long‐term monitoring, motion artifacts and limited spatial resolution can hinder decoding accuracy, particularly in the context of ambulatory rehabilitation.^[^ ^15^ ^]^ Consequently, research efforts have pivoted toward multimodal paradigms, aiming to harness complementary neurophysiological signals to enhance system robustness.^[^ ^16^, ^17^, ^18^, ^19^ ^]^ On this basis, hybrid BCIs that assess both EEG signals and hemodynamic activity have attracted attention. Hemodynamic parameters can be collected through positron emission tomography (PET), functional magnetic resonance imaging (fMRI), and functional near‐infrared spectroscopy (fNIRS). Among these, only fMRI and fNIRS can be recorded simultaneously with EEG. However, although fMRI offers high spatial resolution, its large size and high operating costs limit its widespread BCI usage. fNIRS is a noninvasive spectroscopic approach that is mounted on the scalp, monitoring changes in oxygenated (HbO) and deoxygenated hemoglobin (HbR) via differential near‐infrared light absorption, thereby indicating local brain activation. During regional activation, increased oxygen consumption and carbon dioxide production raise HbR levels while lowering HbO levels.^[^ ^20^ ^]^ By combining the real‐time electrical brain activity tracking of EEG with the high spatiotemporal representation of blood flow of fNIRS, hybrid EEG‐fNIRS BCIs capture immediate motor planning signals alongside extended cortical engagement.^[^ ^21^ ^]^ While this methodological synergy offers a promising approach, it requires sophisticated feature fusion and classification strategies to optimally and effectively integrate the resultant heterogeneous neurophysiological information.

Hybrid EEG‐fNIRS BCIs face inherent challenges in temporal synchronization and heterogeneous feature fusion. Current implementations predominantly employ late‐fusion strategies, in which modality‐specific features are manually extracted and concatenated postacquisition. Conventional approaches predominantly rely on engineered EEG features—such as common spatial patterns (CSP) for sensorimotor rhythm decoding—combined with fNIRS descriptors (e.g., mean, kurtosis) for support vector machine (SVM)/linear discriminant analysis classification.^[^ ^22^, ^23^ ^]^ Gao et al. demonstrated this approach by applying CSP to both EEG and fNIRS signals before SVM‐based fusion, achieving limited cross‐modal integration.^[^ ^24^ ^]^ Recent efforts explore deep learning architectures. Sun et al. compared linear fusion, tensor fusion, and pth‐order polynomial fusion (pth‐PF) methods for hybrid signals,^[^ ^19^ ^]^ while Kwa et al. proposed an fNIRS‐guided attention network (FGANet)‐an early‐fusion design that transforms raw EEG‐fNIRS data into 3D tensors with attention mechanisms.^[^ ^16^ ^]^ Additionally, individual variability remains a key concern in BCI research, prompting some EEG‐decoding algorithms to adopt transfer learning to improve cross‐subject generalizability and to ensure signal stability. For example, the multi‐source geometric metric transfer learning algorithm aggregates multiple feature matrices through Euclidean alignment, thereby improving the distance representation between features.^[^ ^25^ ^]^ Lee et al. used both within‐paradigm and cross‐paradigm transfer to control air conditioners using EEG signals, demonstrating the effectiveness of transfer learning between subjects and paradigms.^[^ ^26^ ^]^ However, the application of transfer learning in hybrid EEG‐fNIRS BCIs remains underexplored, highlighting a critical gap for future research. Furthermore, although multimodal MI datasets from normal subjects have enabled progress in EEG‐fNIRS integration, critical gaps persist in addressing ICH pathophysiology.

**Figure 1:**
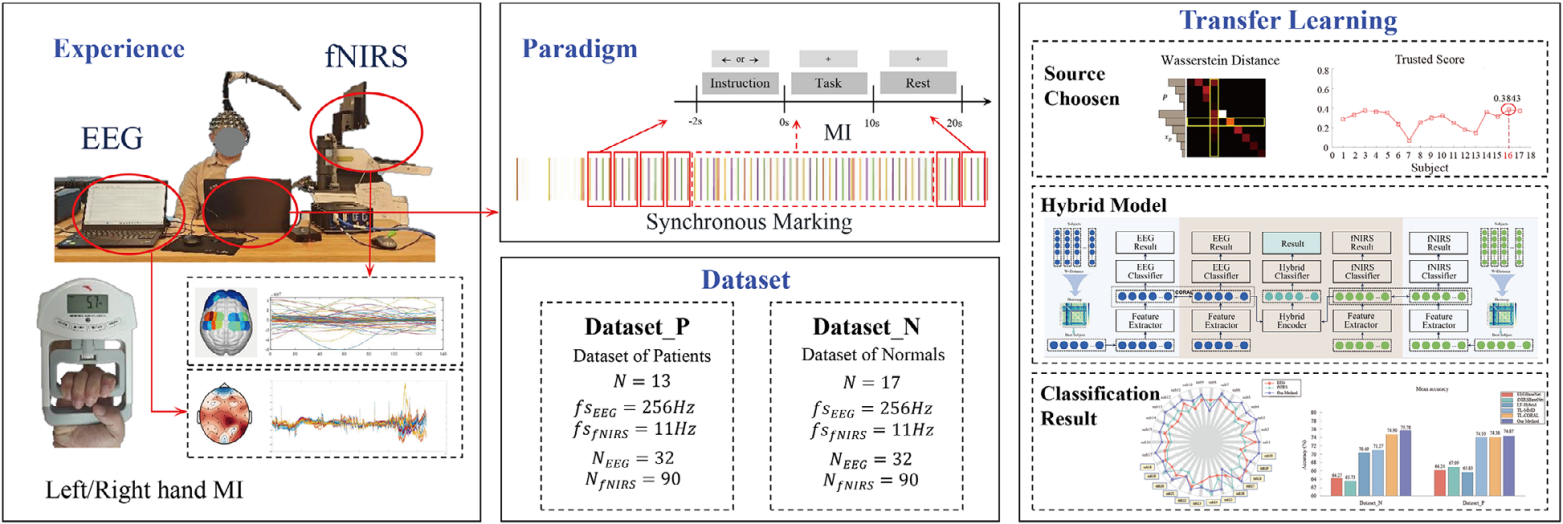
Overview of the study design.

To address the outlined challenges, we collected MI data from both normal subjects and patients with ICH and introduced a transfer learning algorithm for cross‐subject decoding in hybrid BCIs. The detailed experimental workflow is shown schematically in **Figure** **1**. The key contributions of this study include: 1) A multimodal MI dataset comprising normal subjects and patients with ICH; 2) A Wasserstein metric‐based source domain selection method for cross‐subject adaptation; 3) A comprehensive transfer learning framework for hybrid EEG‐fNIRS BCIs.

## 2 Result

### 2.1 Private Data

#### 2.1.1 Time‐Frequency Domain Analysis Between Normal Subjects and Patients with ICH

**Figure 2:**
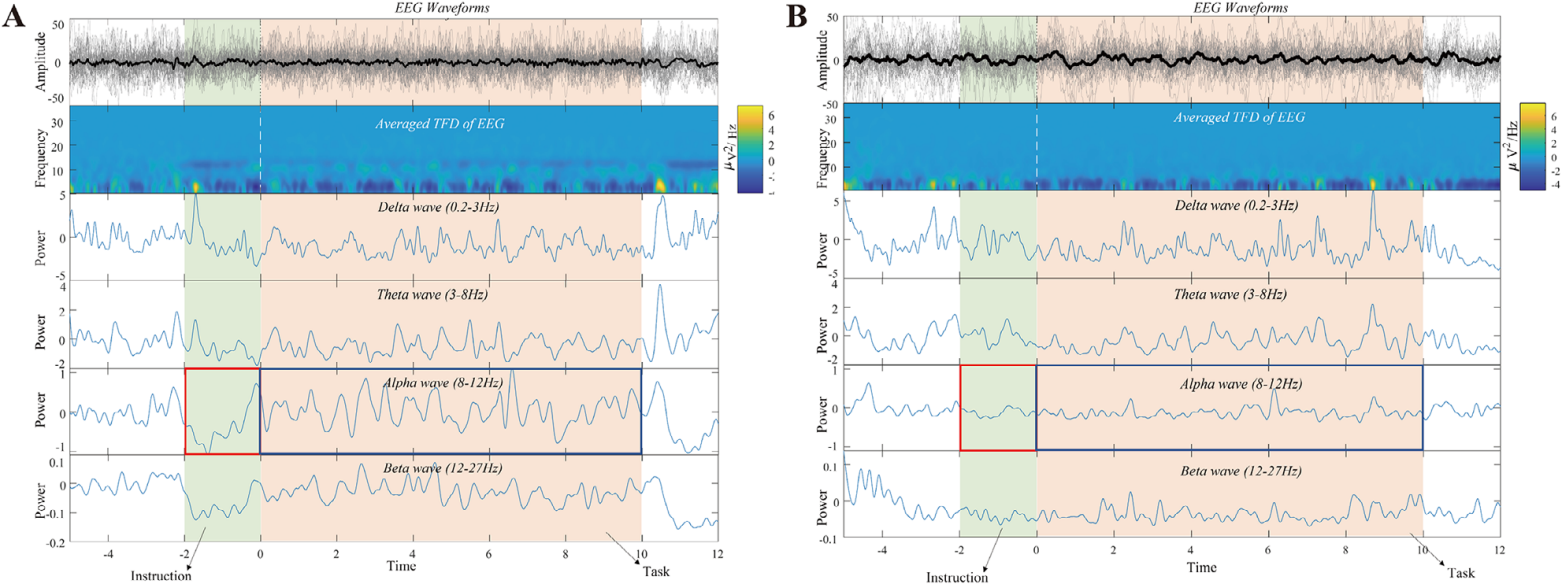
Time‐frequency domain analysis of EEG signals for the normal subject A) and the patient with intracerebral hemorrhage B).

During MI tasks, characteristic EEG rhythm modulation was observed: in the preparation and execution phases of unilateral limb movement, the contralateral sensorimotor cortex exhibited α (8–12 Hz) and β (12–27 Hz) bands, known as event‐related desynchronization (ERD).^[^ ^27^ ^]^ A joint time‐frequency domain analysis was conducted on EEG data from both normal and patient groups. For a representative normal subject (Subject 1), EEG signals from the right sensorimotor area during left‐hand MI trials were selected, and short‐time Fourier transform (STFT) results across 30 trials were averaged. As shown in **Figure** **2**A, the lower four time‐series plots show energy changes across different frequency bands, with green blocks indicating the instruction cue phase and orange‐red blocks indicating the task execution phase. A significant ERD in the α band was observed during the task preparation period. In contrast, the patient data (Subject 21, Patient 4) revealed no significant differences in rhythm modulation characteristics between the instruction and task execution phases (Figure 2B). Clinical assessments indicated left‐limb motor impairment, correlating with reduced α/β desynchronization in contralateral sensorimotor cortex activity (**Table** **1**).

**Table 1:** Basic information of enrolled patients with intracerebral hemorrhage. FMA‐UE, the Fugl Meyer Assessment for Upper Extremities; MBI, the modified Barthel Index; mRS, the modified Rankin scale.

Patient ID	Gender	Age	Lesion Location	FMA‐UE (Unaffected /impaired side)	MBI	mRS
1	Male	68	Right basal ganglia	60/60	85	1
2	Male	48	Right basal ganglia	60/12	55	3
3	Male	35	Left frontal and basal ganglia	30/2	7	4
4	Female	36	Right basal ganglia	30/3	64	4
5	Male	51	Left basal ganglia	34/17	77	1
6	Male	63	Right hypothalamus	24/0	32	4
7	Female	49	brain stem infarction	34/25	95	1
8	Male	68	Left basal ganglia	31/56	95	1
9	Male	36	Right basal ganglia	28/66	97	1
10	Male	46	Right basal ganglia	66/48	98	1
11	Male	60	Left basal ganglia	56/12	43	4
12	Male	47	Left basal ganglia	66/12	64	3
13	Male	39	Left basal ganglia	66/10	81	1

#### 2.1.2 Performance of Multimodal Fusion Model in Normal Subjects and Patients with ICH

**Figure 3:**
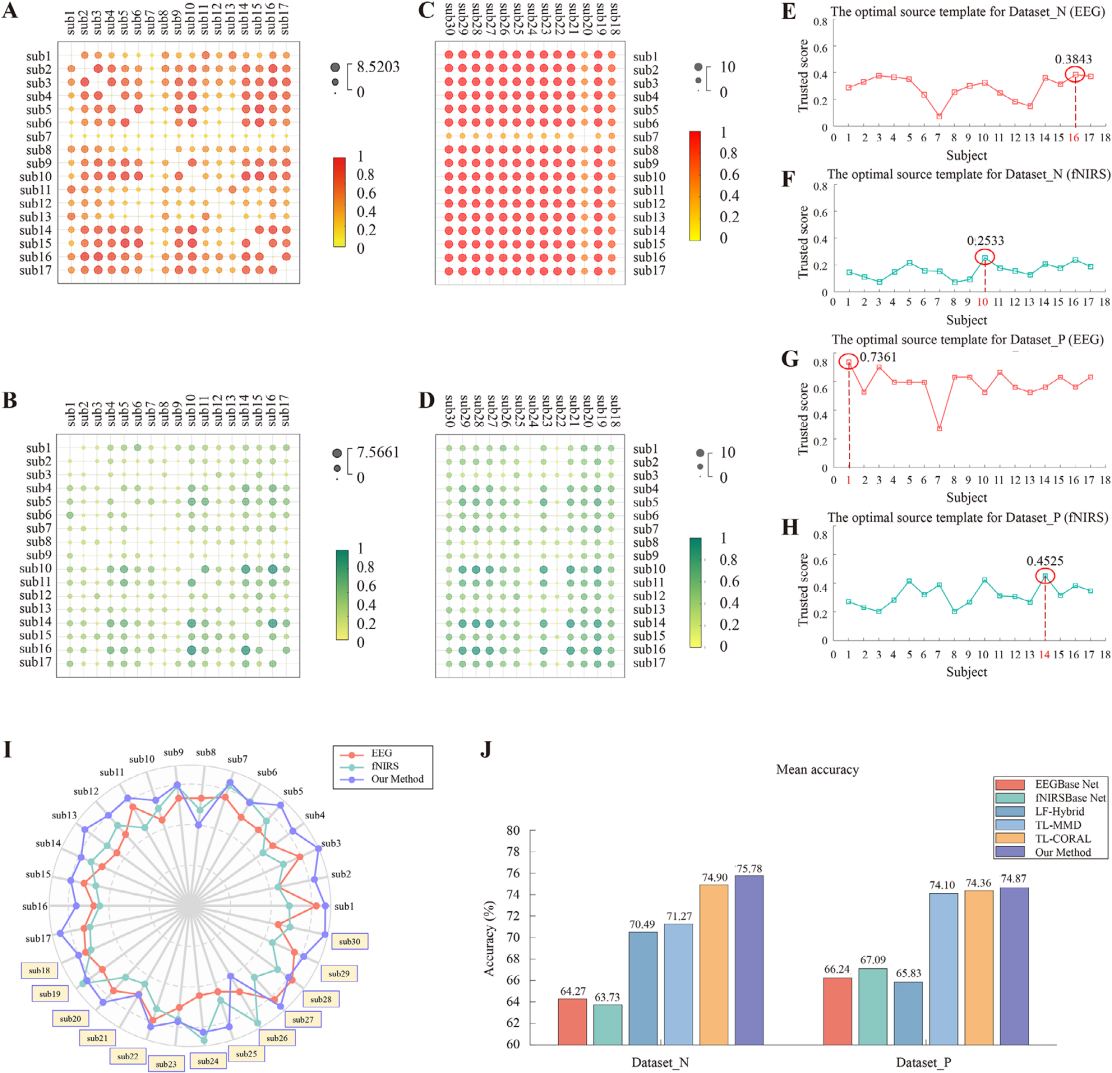
A) Inter‐subject Wasserstein distance heatmap for EEG modality in Dataset_N; B) Inter‐subject Wasserstein distance heatmap for fNIRS modality in Dataset_N; C) Inter‐subject Wasserstein distance heatmap for EEG modality in Dataset_P; D) Inter‐subject Wasserstein distance heatmap for fNIRS modality in Dataset_P; E) Optimal source template for Dataset_N (EEG); F) Optimal source template for Dataset_N (fNIRS); G) Optimal source template for Dataset_P (EEG); H) Optimal source template for Dataset_P (fNIRS); I) Radar plot of motor imagery classification accuracy for each subject; J) Mean classification accuracy in Dataset_N and Dataset_P across modalities and methods.

To enhance MI classification accuracy and feature distinctiveness in patients with ICH, models were first trained using data from normal subjects with robust neural profiles, with the best performers serving as source domains for transfer learning. The private dataset was divided into two subsets: normal subjects (Dataset_N) and patients (Dataset_P). Optimal source domain selection was conducted independently for each modality (EEG and fNIRS) using a Wasserstein metric‐based framework that integrated distribution divergence and classification accuracy (**Figure** **3**A–H).

The highest‐trusted subjects were selected as source domains: Subject 16 (EEG) and Subject 10 (fNIRS) from Dataset_N for normal‐to‐normal transfer, and Subject 1 (EEG) and Subject 14 (fNIRS) from Dataset_N for normal‐to‐patient transfer. Individual subject accuracies were visualized using radar plots (Figure 3I), demonstrating inter‐subject performance variations across experimental conditions. Five‐fold cross‐validation demonstrated that transfer learning improved mean classification accuracy in both Dataset_N and Dataset_P (Figure 3J). Specifically, in Dataset_P, transfer learning yielded a 9.04% increase in mean accuracy compared to baseline methods‐EEGBase Net (66.24%), fNIRSBase Net (67.09%), and the hybrid approach using linear fusion (65.83%). In addition, our transfer learning markedly reduced the performance gap between normal subjects and patients across all modalities: from 1.97% (EEGBase Net), 3.36% (fNIRSBase Net), and 4.66% (linear fusion) to a unified 0.91% post‐transfer. This convergence indicates that the proposed method effectively captures cross‐subject neurodynamic invariants during MI, thereby enhancing model robustness across heterogeneous populations.

#### 2.1.3 Ablation Experiment

**Figure 4:**
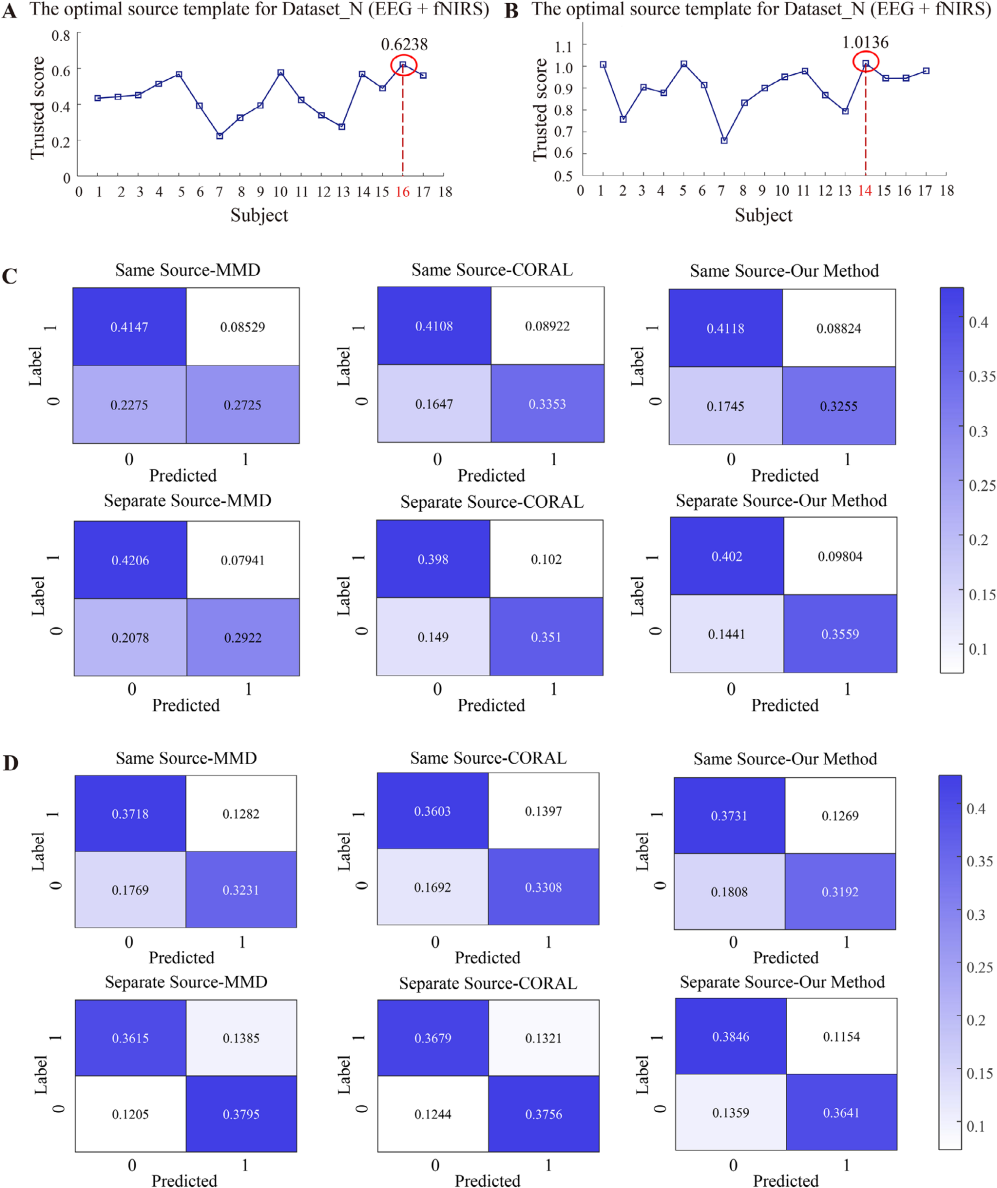
A) Optimal source template for Dataset_N (EEG + fNIRS); B) Optimal source template for Dataset_P (EEG + fNIRS); C) Confusion Matrix of Dataset_N; D) Confusion Matrix of Dataset_P.

To evaluate the effectiveness of the proposed methodological framework—which incorporates both a source domain selection strategy and a transfer learning approach—we conducted comparative experiments using various source domain selection methods (specifically, identifying the optimal single‐subject source domain by aggregating two‐modal trust scores, **Figure** **4**A,B) and established transfer learning techniques, including maximum mean difference (MMD) and CORAL.^[^ ^28^, ^29^ ^]^ Based on a comprehensive analysis of the confusion matrices, as well as the computed accuracy, precision, recall, and F1‐score, we observed that selecting distinct source domains for each modality, as opposed to using a shared source domain, yields improved performance across different transfer methods (Figure 4C,D). Notably, in Dataset_P, an improvement of more than 5% was achieved. Furthermore, the proposed transfer learning framework demonstrates superior performance compared to other existing transfer learning approaches (Figure 3J and **Tables** **2**–**3**). A Friedman test was conducted to assess the statistical significance of the performance differences across methods, yielding *p* < 0.01, indicating statistically significant differences in classification performance among the compared approaches.

**Table 2:** Evaluation metrics of Dataset_N.

Method	Accuracy	Precious	Recall	F1‐Score
Same Source‐MMD	68.73 ± 12.84	80.78 ± 29.26	69.64 ± 16.01	73.80 ± 10.62
Separate Source‐MMD	71.27 ± 14.03	82.61 ± 24.49	72.42 ± 16.68	75.05 ± 11.86
Same Source‐CORAL	74.61 ± 10.99	80.99 ± 21.98	74.16 ± 13.05	76.63 ± 9.17
Separate Source‐CORAL	74.90 ± 11.18	78.47 ± 23.97	77.75 ± 16.26	76.36 ± 9.76
Same Source Our‐Method	73.73 ± 10.73	80.57 ± 24.16	74.40 ± 14.75	75.65 ± 11.15
Separate Source‐Our Method	75.78 ± 11.48	78.78 ± 24.71	78.22 ± 15.32	76.73 ± 11.75

**Table 3:** Evaluation metrics of Dataset_P.

Method	Accuracy	Precious	Recall	F1‐Score
Same Source‐MMD	69.49 ± 11.25	71.79 ± 31.06	75.53 ± 17.95	71.29 ± 12.61
Separate Source‐MMD	74.10 ± 11.24	70.66 ± 25.30	82.03 ± 16.75	73.85 ± 11.55
Same Source‐CORAL	69.10 ± 10.81	69.29 ± 30.21	75.69 ± 17.56	69.25 ± 13.45
Separate Source‐CORAL	74.36 ± 11.63	70.66 ± 29.61	82.20 ± 16.87	74.83 ± 12.06
Same Source Our‐Method	69.23 ± 11.60	71.68 ± 31.71	75.01 ± 17.66	70.76 ± 13.45
Separate Source‐Our Method	74.87 ± 11.65	75.42 ± 25.44	80.34 ± 16.61	76.04 ± 11.00

### 2.2 Public Data

#### 2.2.1 Performance of Multimodal Fusion Model in MI and Mental Arithmetic (MA) Dataset

**Figure 5:**
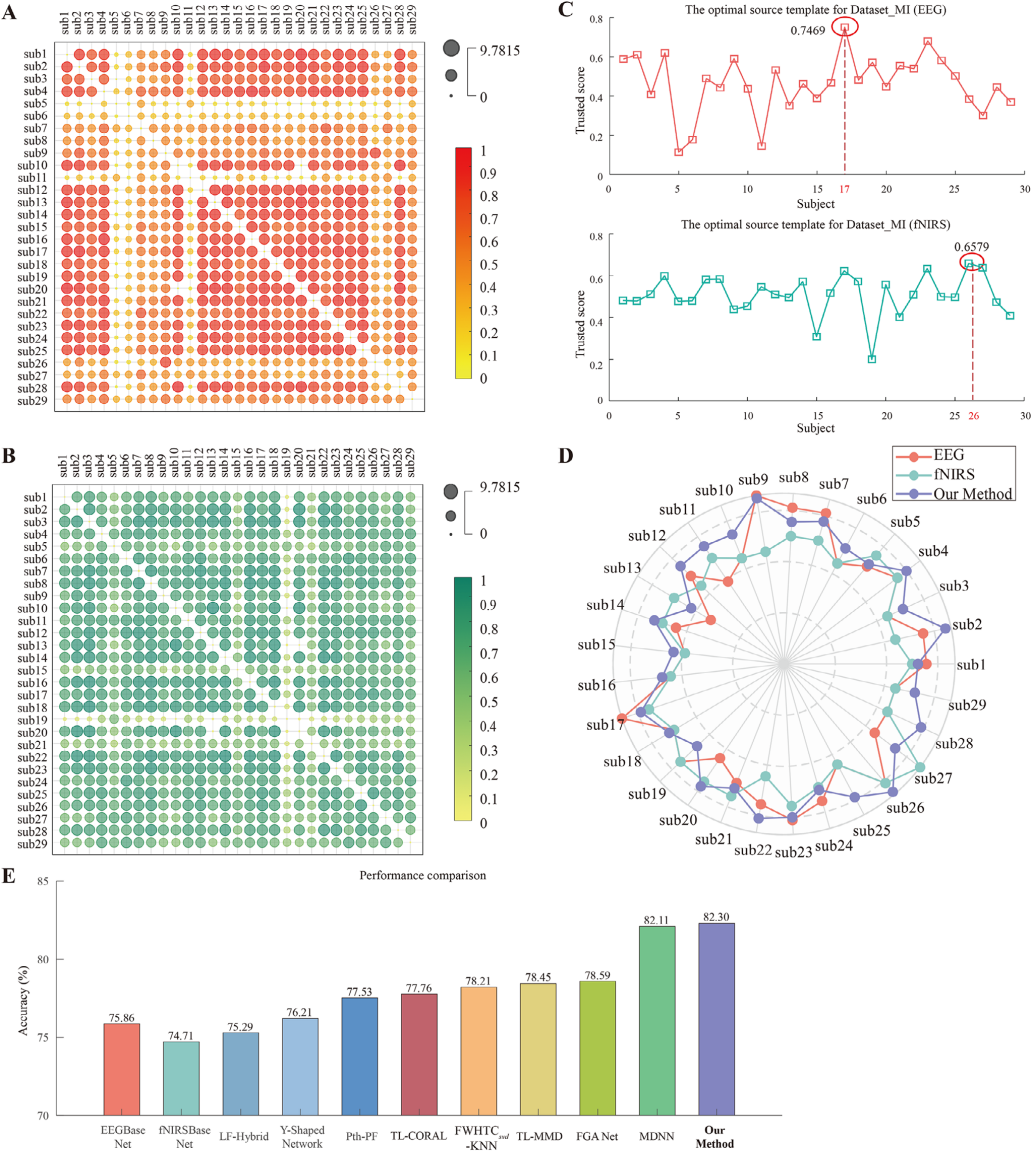
A) Inter‐subject Wasserstein distance heatmap for EEG modality in Dataset_MI; B) Inter‐subject Wasserstein distance heatmap for fNIRS modality in Dataset_MI; C) Optimal source template for the Dataset_MI (EEG and fNIRS); D) Radar plot of motor imagery classification accuracy for each subject; E) Performance comparison between the proposed method and related studies. Pth‐PF: pth‐order polynomial fusion; FWHTC*_svd_*: the singular value decomposition values of the Fast Walsh‐Hadamard transform coefficients; KNN: k‐nearest neighbor algorithm.

**Figure 6:**
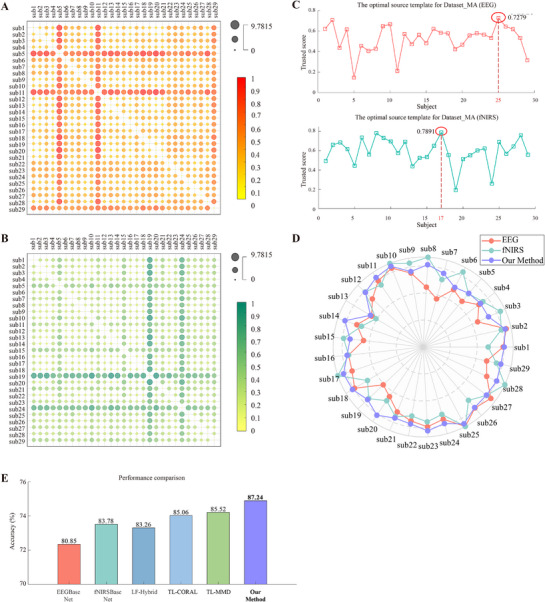
A) Inter‐subject Wasserstein distance heatmap for EEG modality in Dataset_MA; B) Inter‐subject Wasserstein distance heatmap for fNIRS modality in Dataset_MA; C) Optimal source template for the Dataset_MA (EEG and fNIRS); D) Radar plot of motor imagery classification accuracy for each subject; E) Performance comparison between the proposed method and related studies.

To further evaluate the generalization and robustness of the proposed algorithm, publicly available MI dataset and MA dataset were used for external validation. **Figure** **5**A–C illustrate the results of optimal source subject selection, with Subject 17 (EEG) and Subject 26 (fNIRS) identified as the optimal source domains of the MI dataset, and **Figure** **6**A–C shows that subject 25 (EEG) and Subject 17 (fNIRS) were identified as the optimal source domains of MA dataset. Comparative analysis of classification performance using single‐modality models versus the transfer learning framework is shown via radar plots (Figures 5D and 6D), demonstrating that the majority of subjects experienced improved decoding accuracy with the proposed method.

As shown in Figure 5E, for the MI dataset, the proposed transfer learning framework achieved a mean accuracy of 82.30%, thereby outperforming EEGBase Net (75.86%), fNIRSBase Net (74.71%), and linear fusion (75.29%). Additionally, comparisons with existing hybrid EEG‐fNIRS methods on the same open‐source dataset revealed the superior performance of the proposed method: Y‐shaped network (76.21%),^[^ ^30^ ^]^ pth‐PF (77.53%),^[^ ^19^ ^]^ the singular value decomposition values of the Fast Walsh–Hadamard transform coefficients with k‐nearest neighbor algorithm (FWHTC*_svd_‐*KNN) (78.21%),^[^ ^31^ ^]^ FGANet (78.59%),^[^ ^16^ ^]^ and MDNN(82.11%).^[^ ^32^ ^]^

#### 2.2.2 Ablation Experiment

**Figure 7:**
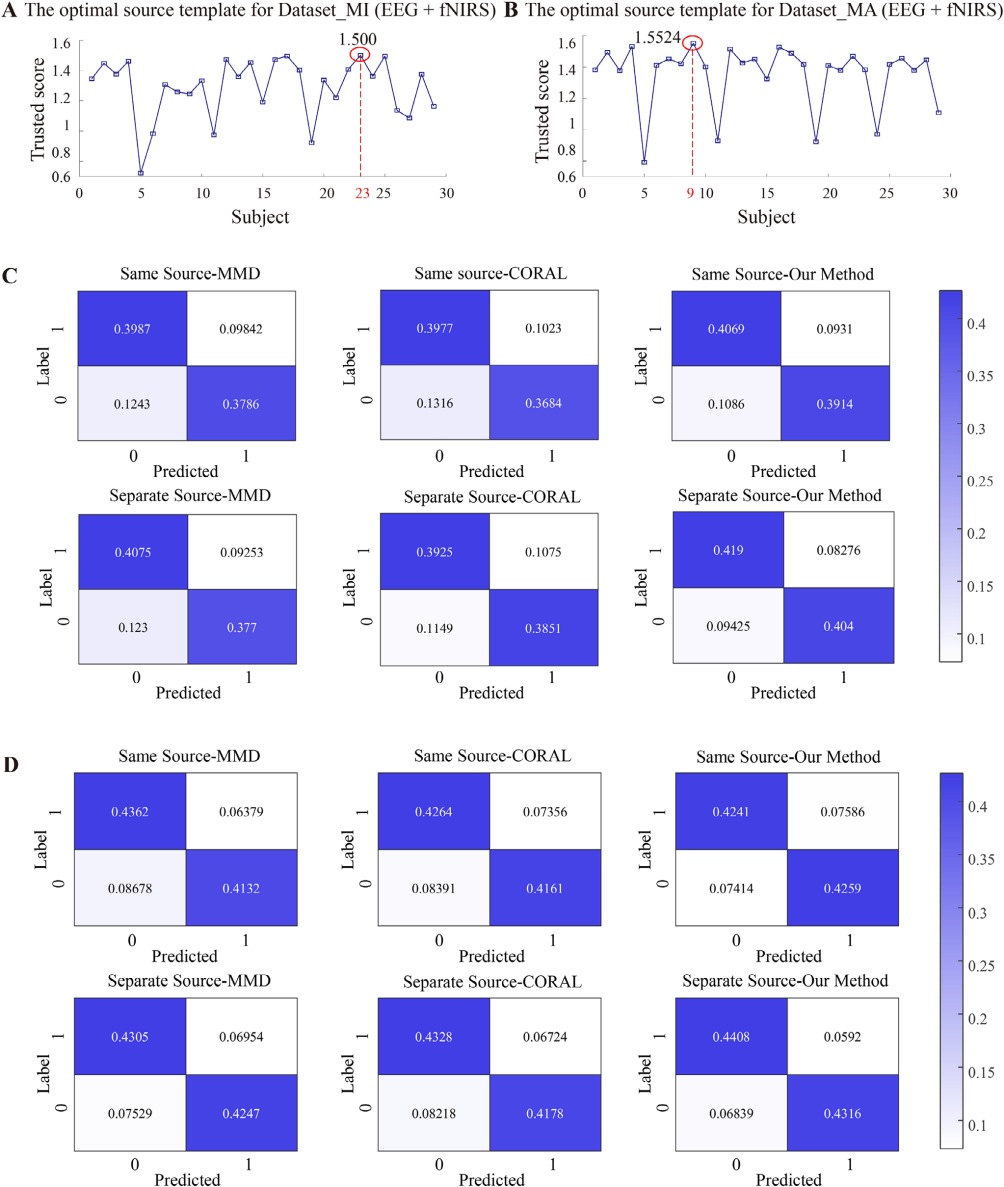
A) Optimal source template for Dataset_MI (EEG + fNIRS); B) Optimal source template for Dataset_MA (EEG + fNIRS); C) Confusion Matrix of Dataset_MI; D) Confusion Matrix of Dataset_MA.

We also adopted the same participant selection method and applied two transfer learning approaches (MMD and CORAL) in conjunction with the proposed framework for comparative analysis. For the MI dataset, subject 23 was selected as the source domain(**Figure** **7**A), while for the MA dataset, subject 9 served as the source domain (Figure 7B). Confusion matrices were computed for each subject, and the results were further validated using fivefold cross‐validation. As shown in Figure 7C,D, both modalities demonstrate improved transfer learning performance when an appropriate source domain is selected. Furthermore, the transfer learning method proposed in this paper exhibits superior performance (Figures 5, 6, and **Tables** **4**, **5**). The statistical analysis confirmed these findings with *p* < 0.01, demonstrating the robustness of the proposed approach.

**Table 4:** Evaluation metrics of Dataset_MI.

Method	Accuracy	Precious	Recall	F1‐Score
Same Source‐MMD	77.73 ± 13.21	77.69 ± 27.61	81.84 ± 16.99	77.71 ± 15.54
Separate Source‐MMD	78.45 ± 12.63	78.54 ± 27.94	81.26 ± 15.73	79.10 ± 14.31
Same Source‐CORAL	76.61 ± 13.82	75.81 ± 31.48	81.10 ± 17.03	77.34 ± 16.30
Separate Source‐CORAL	77.76 ± 12.84	74.86 ± 31.19	81.63 ± 15.64	78.34 ± 15.20
Same Source Our‐Method	79.83 ± 12.13	78.64 ± 26.55	82.79 ± 14.82	78.99 ± 15.28
Separate Source‐Our Method	82.30 ± 11.53	80.90 ± 24.90	84.74 ± 14.32	82.83 ± 12.54

**Table 5:** Evaluation metrics of Dataset_MA.

Method	Accuracy	Precious	Recall	F1‐Score
Same Source‐MMD	84.94 ± 10.78	86.03 ± 20.96	85.62 ± 12.91	84.40 ± 13.15
Separate Source‐MMD	85.52 ± 11.07	84.47 ± 22.20	87.19 ± 12.39	84.81 ± 13.33
Same Source‐CORAL	84.25 ± 11.58	83.22 ± 23.70	86.57 ± 13.22	83.29 ± 14.46
Separate Source‐CORAL	85.06 ± 11.45	85.09 ± 22.17	86.36 ± 12.90	84.73 ± 13.39
Same Source Our‐Method	85.00 ± 11.89	83.41 ± 22.23	87.62 ± 13.80	84.33 ± 13.51
Separate Source‐Our Method	87.24 ± 10.59	86.24 ± 20.12	89.89 ± 12.86	86.71 ± 11.77

## 3 Discussion

The evolution of BCI systems has advanced through the development of both novel hardware platforms and algorithms. While unimodal BCIs are often limited by low signal‐to‐noise ratios and susceptibility to artifacts, hybrid systems address these limitations by integrating complementary neural signals. For instance, hybrid EEG‐fNIRS BCIs combine the millisecond‐level temporal resolution of EEG with the spatial mapping of hemodynamic activity offered by fNIRS. In addition to gains in performance, this multimodal integration enhances interpretability. For example, the spatial information provided by fNIRS signals helps locate specific brain regions, thereby explaining the changes observed in EEG signals and enabling researchers to better understand the relationships between different neural activities and brain regions.

MI has demonstrated clinical efficacy in poststroke upper‐limb rehabilitation, including in ICH, as supported by randomized controlled trials and guideline recommendations.^[^ ^33^, ^34^, ^35^ ^]^ However, the clinical implementation of BCI still faces significant barriers, including ethical considerations and technical obstacles, leading to a relative lack of publicly available multimodal datasets, particularly those involving neurologically impaired individuals. This limitation is exemplified by currently available datasets, such as Shin's multimodal MI dataset, which includes only normal subjects.^[^ ^36^ ^]^ In fact, BCI data from ICH patients may differ from that of normal subjects due to factors such as brain injury, potential changes in cognitive and psychological functions, and older age. Although some stroke datasets have recently emerged, Liu et al. released the first open dataset for left‐ and right‐hand MI tasks in acute stroke patients, which includes unimodal EEG data from 50 patients with cerebral ischemia rather than ICH.^[^ ^37^ ^]^ Additionally, Isaev et al. published the first public dataset containing fNIRS recordings from stroke patients, including 15 individuals with unilateral cortical lesions who completed MI tasks across three separate BCI sessions using different mental tasks.^[^ ^38^ ^]^ However, both datasets are unimodal. Given these limitations, we believe that this study introduces the first multimodal MI dataset that includes ICH patients, providing a novel resource for investigating neuroplasticity mechanisms involved in MI‐BCI‐mediated rehabilitation.

In the field of BCIs, inter‐subject variability stands out as one of the most pressing challenges. Neural signals differ significantly between individuals, necessitating repeated calibration for each subject—a process that hinders the practical application of BCIs. This variability arises from factors such as age, cognitive ability, emotional state, and the quality of the BCI device used. Even within the same participant, signal inconsistencies can occur across sessions due to the nonstationary nature of neural signals, requiring recalibration of the BCI system.^[^ ^39^ ^]^ Studies have shown that variability in BCI performance is more pronounced in patient populations than in healthy individuals.^[^ ^40^, ^41^ ^]^ In ICH, the problem may be more pronounced due to differences in the extent of brain injury among patients. Additionally, research by Mrone et al. identified a strong correlation between motivation and BCI performance,^[^ ^42^ ^]^ and subsequent studies have shown that this relationship is closely related to motor recovery.^[^ ^43^ ^]^ Therefore, in neural rehabilitation for ICH, the lengthy calibration process may undermine patient confidence and motivation, potentially impeding the recovery process. Transfer learning has attracted significant attention as a strategy to address inter‐subject signal variability by updating models to adapt to new users.^[^ ^44^, ^45^, ^46^ ^]^ However, this approach has some limitations. It requires a substantial amount of data from the target object and requires model updates for each new target object. Moreover, there remains a relative lack of research evidence on high‐performance inter‐subject transfer learning models for hybrid EEG‐fNIRS BCIs currently.

Decoding accuracy is another key challenge in the field of hybrid EEG‐fNIRS BCIs. The extraction of features from BCI signals, particularly noninvasive signals, is challenging and highly susceptible to noise. In this context, machine learning and deep learning techniques that do not rely on expert‐designed or manual features are becoming increasingly important for feature extraction and BCI decoding. These methods can effectively handle the high dimensionality and complexity of neural signals, allowing for the automatic extraction of relevant deep features, the mitigation of noise effects, and improvements in overall classification accuracy. Li et al. proposed a Y‐shaped neural network to evaluate the classification performance of EEG‐fNIRS fusion at different stages. Their results show that early fusion outperformed mid‐ and late fusion, achieving an accuracy rate of 76.21% using leave‐one‐out cross‐validation.^[^ ^30^ ^]^ Sun et al. proposed three fusion approaches (linear fusion, tensor fusion, and pth‐PF) and found that the hybrid BCI system outperformed unimodal systems, with pth‐PF delivering the highest MI classification accuracy of 77.53%.^[^ ^19^ ^]^ Ergun et al. extracted and classified features using FWHTC*_svd_‐*KNN, reporting a 6.75% improvement in performance over EEG‐only models, with an accuracy of 78.21% on MI tasks.^[^ ^31^ ^]^ Kwak et al. developed FGANet, an early fusion structure based on deep learning. In this model, 1D multi‐channel EEG and fNIRS signals were transformed into 3D tensors to achieve spatial alignment. A joint representation was then extracted using an fNIRS‐guided attention layer, which generated attention maps to provide more reliable EEG decoding. These attention maps were optimized via spatial correlation regularization, and the EEG prediction score was incorporated into the final output to compensate for the inherent latency of fNIRS signals. For MI tasks, FGANet achieved an accuracy of 78.59%.^[^ ^16^ ^]^ All of the discussed methods were tested on Shin's dataset.^[^ ^36^ ^]^ However, there is currently a lack of research on MI decoding using hybrid EEG‐fNIRS BCI data from individuals with ICH. Meanwhile, most traditional machine learning and deep learning methods require large volumes of labeled data to train high‐performance models. However, in clinical applications, collecting such labeled datasets is time‐consuming, expensive, and often impractical to achieve. To address this limitation, few‐shot learning techniques have been adopted to improve BCI system adaptability using minimal subject‐specific data.^[^ ^47^ ^]^

To address the aforementioned issues, this study proposes a transfer learning framework for MI decoding using hybrid EEG‐fNIRS BCIs. This framework effectively enhances cross‐subject information utilization and decoding accuracy, facilitating its practical application in small‐sample datasets. Experimental results show that the proposed method delivers average MI classification accuracies of 74.72% in ICH patients, 76.96% in normal subjects, and 82.30% in public MI datasets comprising normal subjects, outperforming the existing methods previously discussed. Results on public MA datasets further demonstrate the generalizability of this transfer learning framework. Notably, ablation experiments across all datasets verified that selecting distinct source domains for each modality outperforms using a shared source domain. Importantly, other models employing similar source domain selection strategies would likewise benefit from this methodology.

Although this study has achieved promising results, several important areas warrant investigation. From a research perspective, the relatively small number of ICH patients in this study limits our ability to fully assess the effectiveness and generalizability of the proposed algorithm in this population. At the technical level, challenges remain in addressing more complex cross‐domain tasks—particularly when only a few target‐domain samples are available—as well as in implementing real‐time signal processing and control in hybrid brain‐computer interfaces. From a developmental perspective, the development of hybrid BCIs must consider the relevant ethical and social implications of these methods. Particularly in medical applications, protecting user privacy, ensuring data security, and mitigating potential risks of misuse are all critical issues that demand careful consideration. Furthermore, building upon this study's findings, clinical interventions integrating MI with neurofeedback and other rehabilitation approaches should be advanced promptly to validate and maximize the clinical significance of these outcomes.

## 4 Conclusion

This study presents a novel hybrid EEG‐fNIRS BCI algorithm that integrates multimodal fusion and transfer learning to substantially enhance cross‐domain adaptability and classification accuracy. By employing a Wasserstein metric‐driven source domain selection method, the algorithm effectively minimizes distributional divergence between source and target domains, thereby improving target domain performance. Additionally, the multimodal learning strategy for combining EEG and fNIRS signals enhances the model's adaptability to diverse brain‐activity patterns and improves classification robustness. Together, these advancements provide new opportunities for facilitating upper‐limb motor function and recovery in patients with ICH.

## 5 Experimental Section

### Dataset 1‐Private Data

A total of 30 subjects participated in this study, comprising 17 normal controls (right‐handed, 12 males and 5 females, aged 23.6 ± 1.8 years), and 13 patients with ICH (right‐handed, 11 males and 2 females, aged 49.7 ± 11.38 years). The time since ICH onset ranged from 2 days to 2 months (Table 1). The study protocol was approved by the Ethics Committee of Tongji Hospital, Tongji Medical College, Huazhong University of Science and Technology (approval number: TJ‐IRB202412123) and was registered in the National Medical Research Registration and Filing Information System of China (No. MR‐42‐25‐008403).

**Figure 8:**
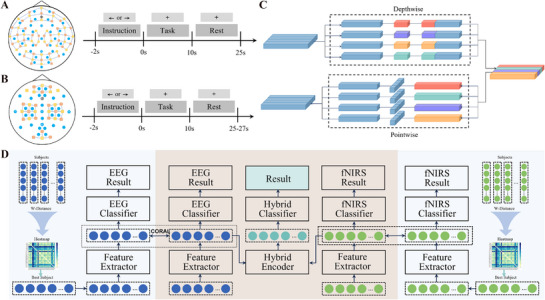
A) Channel configuration and experimental paradigm for the private dataset; B) Channel configuration and experimental paradigm for the public motor imagery dataset; C) Schematic diagram of depthwise separable convolution; D) Architecture of the multimodal fusion model.

As illustrated in **Figure** **8**A, subjects wore a customized integrated cap that synchronously recorded 32‐channel EEG and 90‐channel fNIRS signals. Temporal synchronization across modalities was achieved using E‐Prime 3.0 event markers (Psychology Software Tools Inc., USA), which simultaneously triggered both recording systems during experimental paradigms. The EEG signals were acquired via a g.HIamp amplifier (g.tec medical engineering, Graz, Austria) at a sampling frequency of 256 Hz. Concurrently, a continuous‐wave multichannel fNIRS device (NirScan, Danyang Huichuang Medical Equipment Co. Ltd., Danyang, China) monitored real‐time oxygenation changes during the MI task. The fNIRS system emitted near‐infrared lasers at 760 and 850 nm with an 11 Hz sampling rate. The distance between each source and detector was 3 cm. On the day of data collection, subjects were instructed to abstain from consuming any caffeinated or alcoholic beverages and to avoid strenuous activity to minimize external influences over brain activity. None of the subjects had prior experience with the experimental tasks or the equipment used in the study. During recording, participants were seated ≈25 cm from a monitor with their heads stabilized to limit motion artifacts. Each trial commenced with an auditory cue that was synchronized to a blue‐background screen displaying a central yellow fixation cross. A directional arrow (left/right) then appeared for 2s, prompting the respective hand MI task for 10s, followed by a 15‐s rest indicated by a short beep and a return to the fixation cross. Each subject performed 30 trials of MI for each hand, for a total of 60 trials, arranged into two sessions (15 trials per hand per session), separated by a 120‐s rest interval. The directions of the arrows were counterbalanced across participants in an effort to reduce bias in task presentation.

### Dataset 2‐Public MI Data

This study additionally used a publicly accessible multimodal MI dataset published by Shin et al.^[^ ^36^ ^]^ A total of 29 subjects were involved (28 right‐handed, 1 left‐handed, 14 males, and 15 females, aged 28.5 ± 3.7 years). EEG data were collected via a 30‐channel BrainAmp EEG amplifier (Brain Products GmbH, Gilching, Germany) at a sampling rate of 1000 Hz, while 36‐channel fNIRS signals were collected using a NIRScout system (NIRx GmbH, Berlin, Germany) at a 12.5 Hz sampling rate. As shown in Figure 8B, each trial consisted of three segments: a 2‐s instruction period, a 10‐s MI task, and a 15‐to‐17‐s rest period. On session included a 60‐s pre‐rest, 20 repetitions of the above trial (10 left‐hand MI and 10 right‐hand hand‐MI in random order), and a 60‐s pos‐trest. Each subject completed three sessions, yielding a total of 60 MI trials.

### Dataset 3‐Public MA Data

A MA dataset also published by Shin et al. is used.^[^ ^36^ ^]^ For this task, the number of subjects and the overall structure of the paradigm execution remain consistent with those of the MI dataset, although the nature of the task differs. Specifically, during the 2‐s instruction phase of the paradigm preparation, a simple arithmetic task involving three‐digit minus one‐digit subtraction is presented to the subjects. Throughout the task, a black fixation cross is displayed on the screen as a visual cue, instructing participants to mentally repeat and solve the subtraction problem. In the baseline condition, no specific cognitive load is imposed; only the presentation of the black cross indicates that subjects should rest and remain attentive. All other experimental settings are identical to those used during the acquisition of the MI dataset.

### Preprocessing Process

Prior to data classification, rigorous preprocessing protocols were implemented for both EEG and fNIRS data. For EEG signals, the original datasets with a sampling frequency of 1000 Hz underwent down‐sampling to 200 Hz as the initial processing step. Subsequent band‐pass filtering (0.5–50 Hz) was applied to both the self‐collected and publicly available datasets post‐down sampling to mitigate low‐frequency physiological artifacts (e.g., slow cortical potentials) and high‐frequency noise sources (e.g., electromyographic interference and motion artifacts), while preserving neurophysiologically relevant frequency bands (δ, θ, α, β, γ). To address potential signal drifts arising from systemic or environmental factors during EEG acquisition, baseline correction was performed by subtracting the mean signal value from a 3‐s pre‐stimulus interval preceding experimental tasks, thereby stabilizing signal amplitude references.

For fNIRS signals, preprocessing began with the Beer–Lambert law‐based conversion of raw optical density measurements into hemodynamic parameters (oxy/hemoglobin concentrations). Following this transformation, the data underwent analogous filtering and baseline correction procedures. However, fNIRS‐specific band‐pass filtering (0.01–0.1 Hz) was employed to isolate cerebral hemodynamic responses by suppressing high‐frequency motion artifacts and low‐frequency baseline drifts. The same baseline correction methodology was applied, using the mean signal value from a 3‐s pre‐task interval as the reference for drift removal.

### Time‐Frequency Domain Characterization of EEG Signals

EEG signals are characterized as multi‐channel univariate time series, typically analyzed in either the temporal or spectral domain. Temporal features (e.g., mean amplitude, peak magnitude, signal steepness) exhibit limited reliability due to susceptibility to acquisition noise, making spectral analysis the predominant methodology. In this study, EEG data were bandpass filtered from 0.5 to 40 Hz to isolate physiologically relevant oscillations, then transformed into time‐frequency representations using the STFT:

(1) $$STFT\left(n,k\right)=\sum_{m}^{N-1}x\left(n+m\right)\omega \left(m\right)e^{-j2\pi nm/N}\;$$

where ω(·) denotes the sliding window function and m indexes temporal segments.

STFT‐based spectral power quantification enabled the profiling of energy distributions across canonical frequency bands (δ, θ, α, β, γ). Sliding‐window STFT visualizations revealed dynamic changes in spectral content over time, allowing for preliminary tracking of neurodynamic patterns during the motor imagery task. These electrophysiological profiles were subsequently validated against experimental event markers using neurobiological interpretative frameworks.

### Single‐Model Classification of EEG and fNIRS

This study introduced two basic classification networks (EEGBase Net and fNIRSBase Net), each designed to extract modality‐specific features and classify MI tasks. Each network comprises a feature extractor and a classifier, functioning independently on the respective EEG and fNIRS signals. The feature extraction modules contain three strategically designed convolutional layers: the first two utilize 1D convolutions with distinct kernel sizes to capture nonlinear temporal features, while the third applies depthwise separable convolution, integrating depthwise and pointwise operations for efficient inter‐channel processing (Figure 8C). This design delivers three main benefits: increased computational efficiency through parameter reduction,^[^ ^48^, ^49^ ^]^ enhancement of intra‐channel cohesion for multi‐channel signal processing, and increased model compactness. Given the high inter‐channel coupling in neurophysiological data, this architecture processes input signals as multi‐channel 1D time series, streamlining the feature extraction process and facilitating a seamless integration with analytical models.

Convolutional parameters were consistent across both networks: convolutional groups were defined as *F1 = 16* and F2 = F1 * D (D = 4), with kernel sizes scaled to match input sequence lengths. Batch normalization followed each convolutional layer to improve convergence, mitigate vanishing gradients, and enhance generalizability. Feature maps were subsequently reduced via adaptive average pooling, flattened, and then classified using a linear layer. Detailed architectural specifications are presented in **Table** **6**.

**Table 6:** EEGBase Net and fNIRSBase Net structures.

Network	Layer	Input	Operation	Filter size	Padding	Activation	Output
EEGBase Net	Conv1	30*2000	Conv1D	F1 * 63	31	Sigmoid	F1 * 2000
			BathNorm				
	Conv2	F1*2000	Conv1D	F2 * 30	/	Sigmoid	F2 * 1971
			BathNorm				
	Pooling1	F2*1971	Avgpooling	4	/	/	F2 * 492
	Conv3	F2*492	SeprateConv1D	F2 * 15	7	Sigmoid	F2 * 492
			BathNorm				
	Pooling2	F2*492	Avgpooling	2	/	/	F2 * 246
	Flatten	F2*246	/	/	/	/	(F2 * 246)
	Classifier	(F2*246)	Linear	(F2 * 246) * 2	/	/	2
fNIRSBase Net	Conv1	36*100	Conv1D	F1 * 36	18	Sigmoid	F1 * 100
			BathNorm				
	Conv2	F1*100	Conv1D	F2 * 101	50	Sigmoid	F2 * 100
			BathNorm				
	Pooling1	F2*100	Avgpooling	4	/	/	F2 * 25
	Conv3	F2*25	SeprateConv1D	F2 * 15	7	Sigmoid	F2 * 17
			BathNorm				
	Pooling2	F2*25	Avgpooling	2	/	/	F2*17
	Flatten	F2*17	/	/	/	/	(F2*17)
	Classifier	(F2*17)	Linear	(F2 * 17) * 2	/	/	2

### Wasserstein Metric‐Driven Source Domain Selection Method

In multi‐source domain transfer learning, the proper source domain significantly affects the accuracy of the target domain classifier. The most direct approach selects the subject with the highest classification accuracy as the source domain. The higher the accuracy of the source domain, the more efficient and stable the features within its data, and the more dependable the features that can be acquired by the target domain. Moreover, aside from accuracy, a greater similarity between the source domain and the target domain leads to more efficient transfer. Consequently, a source‐domain selection method was developed that integrates both the Wasserstein distances between subjects and their respective validation set accuracies.

The Wasserstein distance is a commonly used metric in deep learning and computer vision for measuring the similarity between histogram distributions.^[^ ^50^ ^]^ It calculates the minimum cost required to transform the histogram of one distribution into that of another. More intuitively, it can be thought of as the minimal cost plan to reshape one pile of earth into another pile (thus, it is also known as the Earth Mover's Distance). The Wasserstein distance can be expressed as:

(2) $$W\left(p,q\right)=\mathrm{in}f_{\gamma ∼\prod \left(p,q\right)}\;E_{x,y∼\gamma }\left[\left|\left|x-y\right|\right|\right]$$

where, *p* and *q* represent two distributions, γ represents the set of all possible joint distributions of *p* and *q*. For each possible γ, sample*s* *x* and *y* are drawn, and the sample distance ||*x* − *y*|| is calculated to determine the expected distance under that joint distribution. The minimum value of this expectation across all *y* is the Wasserstein distance.

To identify the optimal source domain, the pairwise Wasserstein distances were computed among all subjects, construct a similarity matrix, and calculate the average Wasserstein distance for each subject relative to all others. The original data preprocessed of the two models was used to calculate the Wasserstein distance, as described in Section 2.2, which can effectively reduce the influence of noise and other factors. A trusted score is then computed to balance classification accuracy and distributional similarity, allowing to select the subject with both the highest accuracy and the greatest self‐similarity as the source domain. Specifically, the average Wasserstein distance was normalized, computed the complement (1–normalized value), and then multiply by the subject's classification accuracy to yield the trusted score:

(3) $$T_{k}=Acc_{k}\;\times \left(1-Norm\left(\sum_{i=1}^{n}W\left(k,i\right)\right)\right)$$

where, *Acc_k_* represents the classification accuracy of the k‐th subject, and *W*(*k*, *i*) is the Wasserstein distance between the k‐th and i‐th subjects.

During training, the EEG and fNIRS subjects with the highest trusted scores (potentially from different individuals) are selected as the optimal source domains for transfer learning.

### Dynamic Fusion Method for Multimodal Transfer Losses

Since Wasserstein distance was used to guide the selection of source domains, Deep CORAL was employed to compute domain adaptation loss based on statistical distribution differences between source and target domains. Deep CORAL reduces and minimizes domain shift by aligning the covariance matrices of extracted features from both domains, thus reducing divergence in the first and second‐order statistics (mean and covariance).^[^ ^51^ ^]^

(4) $$L_{coral}=\frac{1}{4d^{2}}∥\tilde{F_{s}}-\tilde{F_{t}}{{∥}_{F}}^{2}\;$$

where

(5) $$\tilde{F_{s}}=\frac{1}{n_{s}-1}\;\left({F_{s}}^{T}F_{s}-\frac{1}{n_{s}}{\left(1^{T}F_{s}\right)}^{T}\left(1^{T}F_{s}\right)\right)$$

and

(6) $$\tilde{F_{t}}=\frac{1}{n_{t}-1}\;\left({F_{t}}^{T}F_{t}-\frac{1}{n_{t}}{\left(1^{T}F_{t}\right)}^{T}\left(1^{T}F_{t}\right)\right)$$

these matrices represent the covariance matrices of the source and target domain features. *F_s_* and *F*_t_ denote the source‐domain and target‐domain features, respectively, used for CORAL computation.

In addition, to further align the distributions within the same class across domains, Deep Canonical Correlation Analysis (DCCA) was utilized.^[^ ^52^ ^]^ Canonical Correlation Analysis (CCA), which is closely related to Principal Component Analysis (PCA), performs linear transformations on two sets of variables to maximize their mutual correlation. Unlike PCA, which identifies directions of maximum variance within a single dataset, CCA focuses on uncovering features that represent shared structure across domains.^[^ ^53^ ^]^

By performing feature extraction on both source‐domain and target‐domain data to obtain high‐dimensional abstract features and applying CCA to identify projections with maximum correlation, the alignment was increased between the source and target domains in a shared latent space. When integrated into deep neural networks, CCA can accommodate nonlinear relationships. After nonlinear projection via the deep neural network, a CCA‐based loss function can be used to optimize feature extraction.

Specifically, the goal of CCA is to find the linear subspaces that maximize the correlation between two inputs *F_s_* and *F_t_*:

(7) $$\left(A,B\right)=arg\max_{A,B}corr\left(A,B\right)$$

Let *F_s_* =  *fD_s_*,θ_*f*_ and *F_t_* =  *gD_t_*,θ_*g*_ represent the features after the nonlinear transformation by the neural network, *f* and *g* represent nonlinear optimization functions of the feature extractor in EEGBase Net and fNIRSBase Net, and the θ_*f*_ and θ_*g*_ represent the parameters of *f* and *g*. The centered data matrices are defined as $\bar{F_{s}}=F_{s}\;-\frac{1}{m}F_{s}1$ (the calculation process is the same for $\bar{\;F_{t}}$). CCA aims to find the linear mappings $\left(\omega_{s}^{T}\bar{F_{s}},\omega_{t}^{T}\bar{F_{t}}\right)$ that maximize the correlation:

(8) $$\begin{array}{c} \left(\omega_{s},\omega_{t}\right)=arg\max_{\omega_{s},\omega_{t}}corr\left(\omega_{s}^{T}\bar{F_{s}},\omega_{t}^{T}\bar{F_{t}}\right)=arg\max_{\omega_{s},\omega_{t}}\frac{\omega_{s}^{T}H_{s,t}\omega_{t}}{\sqrt{\omega_{s}^{T}H_{s,s}\omega_{s}}\sqrt{\omega_{t}^{T}H_{t,t}\omega_{t}}} \end{array}$$

where $H_{s,s}=cov\bar{F_{s}}\bar{F_{s}}$ and $H_{t,t}=cov\bar{F_{t}}\bar{F_{t}}$ are the covariance matrices, and $H_{s,t}=cov\bar{F_{s}}\bar{F_{t}}$​ is the cross‐covariance matrix. The total correlation of the top k components of *F_s_* and *F_t_* is the sum of the top k singular values of the matrix T:

(9) $$H_{s,s}^{-\frac{1}{2}}H_{s,t}H_{t,t}^{-\frac{1}{2}}$$

Then the correlation can be computed as:

(10)

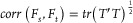




The loss is defined as the negative of this correlation:

(11)

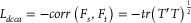




The total transfer loss function is expressed as:

(12) $$L_{trans}=L_{dcca}+L_{coral}$$

The transfer learning process is performed independently on EEG and fNIRS single‐modality data. After feature extraction, a late‐stage fusion step combines these features. The weighting coefficients for each modality are determined based on validation accuracy, thereby optimizing performance in the multimodal classification framework.

The loss of the final classification result is calculated using the cross‐entropy function:

(13) $$L_{clf}=-\frac{1}{N}y_{i}\log\left(y^{pred}\left(X_{i}\right)\right)$$

The final loss function is:

(14) $$L=L_{clf}+aL_{trans\_1}+bF_{trans\_2}$$

where, $L_{trans\_1}$ and $L_{trans\_2}$ denote the transfer losses for EEG and fNIRS, respectively.

### Multimodal Fusion Model‐Optimally Selected Source Domain

To obtain the trusted score for each subject, the single‐modal data must first be trained separately, and all subjects are pre‐trained using the EEGBase Net and fNIRSBase Net.

First, data preprocessing was conducted by segmenting each recording session into valid trials. The resulting single‐modality data were structured as Ls * Lch, indicating the use of raw time‐series data. Here, *Ls* represents the sample length (based on sampling frequency and trial duration), and *Lch* which represents the number of EEG or fNIRS channels. Wasserstein distances between all subject pairs were then calculated for each modality, generating modality‐specific similarity heatmaps. After pretraining each subject's data and recording the classification accuracies, a trusted score was calculated for each subject using the similarity and accuracy metrics. Subjects with the highest trusted scores in each modality were selected as the optimal source domains.

### Multimodal Fusion Model‐Construction and Training of the Fusion Model

Because EEGBase Net and fNIRSBase Net share identical feature extraction module structures, the parameters of the optimal source subject models can be directly transferred and integrated. To mitigate any potential overfitting during the training process, the architecture of the fusion model was simplified. Features extracted from both modalities were concatenated through a direct connection, then passed through a flattening layer followed by a linear layer to map the fused features to the label space to classify the MI tasks. During training, features from both the source domain and the target domain were simultaneously calculated through the network. Based on the calculated DCCA loss and Deep CORAL loss, the transfer losses for the source and target domains were obtained. Assuming that the original single‐modal signal accuracies of the target domain were *A_eeg_* and *A_nirs_*, then the mixing ratio is:

(15) $$F=aF_{eeg-s}+bF_{nirs-s}$$

(16) $$a=\frac{A_{eeg}}{A_{eeg}+A_{nirs}}\;,\;b=\frac{A_{nirs}}{A_{eeg}+A_{nirs}}\;$$

where, *F* is the fused feature, and *F*_*eeg* − *s*_ and *F*_*nirs* − *s*_ are the two kinds of features extracted from the source domain.

The final loss function was defined as a weighted sum of the classification loss and the modality‐specific transfer losses. To improve the propagation efficiency during the training process, the Adam optimizer was employed for backpropagation, with a learning rate set to 0.001. This helped to reduce overfitting of the model and improve its generalizability. The specific training steps are provided in Figure 8D and **Table** **7**.

**Table 7:** Detailed training steps for the proposed hybrid EEG‐fNIRS BCI framework.

Steps	Description
1	Train the EEG data of all subjects using the EEGBase Net, save the trained models, and record the results.
2	Train the fNIRS data of all subjects using fNIRSBase Net, save the trained models, and record the results.
3	Calculate the Wasserstein distance heatmap for the EEG data of all subjects, compute each subject's trusted score, and select the optimal subject in the EEG modality.
4	Calculate the Wasserstein distance heatmap of the fNIRS data of all subjects, compute each subject's trusted score, and select the optimal subject in the fNIRS modality.
5	Select the feature extractors of the optimal subjects in the EEG and fNIRS modalities to construct a fusion classification model.
6	Use the optimal EEG and fNIRS subjects as source‐domain data, and the remaining subjects as target‐domain data. Apply DCCA loss and Deep CORAL for transfer loss, and cross‐entropy loss for classification.
7	Train the model, then evaluate it to obtain the final classification results.

### Statistical Analysis

Data processing and model construction were implemented using Python 3.10. Models were developed with PyTorch 1.12 and CUDA 11.3, utilizing an Intel i5‐13600K CPU and NVIDIA 4060Ti GPU for training. Evaluation metrics included accuracy, precision, recall, and F1‐Score, presented as mean ± SD. The Friedman test (a non‐parametric method for comparing multiple related samples) assessed significant differences in cross‐validated accuracy across models.

## Conflict of Interest

L.H. is employed by Wuhan Neuracom Technology Development Co., Ltd. All other authors disclose no relevant relationships.

## Data Availability

Further information and data requests should be directed to the corresponding authors.
